# The science of clinical practice: disease diagnosis or patient prognosis? Evidence about “what is likely to happen” should shape clinical practice

**DOI:** 10.1186/s12916-014-0265-4

**Published:** 2015-01-30

**Authors:** Peter Croft, Douglas G Altman, Jonathan J Deeks, Kate M Dunn, Alastair D Hay, Harry Hemingway, Linda LeResche, George Peat, Pablo Perel, Steffen E Petersen, Richard D Riley, Ian Roberts, Michael Sharpe, Richard J Stevens, Danielle A Van Der Windt, Michael Von Korff, Adam Timmis

**Affiliations:** Arthritis Research UK Primary Care Centre, Research Institute for Primary Care and Health Sciences, Keele University, Keele, Staffordshire ST5 5BG UK; Centre for Statistics in Medicine, Nuffield Department of Orthopaedics, Rheumatology and Musculoskeletal Sciences, University of Oxford, Botnar Research Centre, Windmill Road, Oxford, OX3 7LD UK; School of Health and Population Sciences, College of Medical and Dental Sciences, University of Birmingham, Birmingham, B15 2TT UK; Centre for Academic Primary Care, School of Social and Community Medicine, University of Bristol, Bristol, BS8 2PS UK; Farr Institute of Health Informatics Research, London, and UCL Institute of Health Informatics, London, NW1 2DA UK; School of Dentistry Office of Research, Box 357480, University of Washington, Seattle, WA 98195 USA; Epidemiology & Population Health Faculty, London School of Hygiene & Tropical Medicine, London, WC1E 7HT UK; William Harvey Research Institute and NIHR Cardiovascular Biomedical Research Unit at Barts, Queen Mary University of London, London, EC1M 6BQ UK; Public Health, Epidemiology and Biostatistics, School of Health and Population Sciences, University of Birmingham, Birmingham, B15 2TT UK; Oxford Psychological Medicine Research, Department of Psychiatry, University of Oxford, Warneford Hospital, Oxford, OX3 7JX UK; Nuffield Department of Primary Care Health Sciences, University of Oxford, Oxford, OX2 6GG UK; Group Health Research Institute, Group Health Cooperative, Seattle, WA 98101 USA; NIHR Cardiovascular Biomedical Research Unit at Barts, Queen Mary University of London, London, EC1M 6BQ UK

**Keywords:** Clinical decision-making, Contested diagnoses, Diagnosis, Evidence-based medicine, Information, Outcomes of care, Overdiagnosis, Prognosis, Stratified medicine

## Abstract

**Background:**

Diagnosis is the traditional basis for decision-making in clinical practice. Evidence is often lacking about future benefits and harms of these decisions for patients diagnosed with and without disease. We propose that a model of clinical practice focused on patient prognosis and predicting the likelihood of future outcomes may be more useful.

**Discussion:**

Disease diagnosis can provide crucial information for clinical decisions that influence outcome in serious acute illness. However, the central role of diagnosis in clinical practice is challenged by evidence that it does not always benefit patients and that factors other than disease are important in determining patient outcome. The concept of disease as a dichotomous ‘yes’ or ‘no’ is challenged by the frequent use of diagnostic indicators with continuous distributions, such as blood sugar, which are better understood as contributing information about the probability of a patient’s future outcome. Moreover, many illnesses, such as chronic fatigue, cannot usefully be labelled from a disease-diagnosis perspective. In such cases, a prognostic model provides an alternative framework for clinical practice that extends beyond disease and diagnosis and incorporates a wide range of information to predict future patient outcomes and to guide decisions to improve them. Such information embraces non-disease factors and genetic and other biomarkers which influence outcome.

**Summary:**

Patient prognosis can provide the framework for modern clinical practice to integrate information from the expanding biological, social, and clinical database for more effective and efficient care.

## Background

The traditional model of clinical practice incorporates diagnosis, prognosis, and treatment [1]. Diagnosis classifies the sick patient as having or not having a particular disease. Historically, diagnosis was regarded as the primary guide to treatment and prognosis (“what is likely to happen in the future”), and is still considered the core component of clinical practice [2].

This traditional model now has to meet the demand for health care to deliver demonstrable quality. Changes in clinical practice must be justified by better outcomes, as valued by patients, or more efficient and safer delivery of health care. The usefulness of diagnostic and treatment decisions, and the value of new tests and interventions, are judged by whether patients classified with diagnosed disease do better and those classified without disease come to no harm [3].

This requires information about patient prognosis – the likelihood of future outcomes in patients with a given disease or health condition. Prognosis research aims to understand the likelihood of different outcomes, which factors predict these likelihoods, how best to estimate an individual’s likelihood of different outcomes, and how this information can be used to target interventions and improve outcomes [4].

Importantly, patient prognosis is influenced by more than disease diagnosis and diagnosis-driven treatment. Among women with advanced breast cancer, for example, treatment responsiveness and toxicity as well as survival are informed by their physical wellbeing and appetite prior to the start of treatment [5]. The multiplicity of biological, clinical, and social factors that inform the likelihood of an individual’s future outcome challenges the idea that prognosis and treatment selection are exclusively determined by diagnosis. Guidelines on statin use, for example, have shifted from “treat hypercholesterolaemia” to “treat the risk of adverse cardiovascular outcomes”, emphasising the continuous and multifactorial nature of risk for adverse outcomes that might be lowered by intervention [6,7].

In this paper, we consider the case for prognosis to replace diagnosis as the framework for clinical decision-making. For some patients, especially those with acute illness or injury, a prognosis-based model will be dominated by diagnostic disease-based information. For many others, notably patients with long-term ill-health, healthy people classified as having a risk-based condition, and persons in screening programmes, a model framed by prognosis would integrate diagnostic and treatment information with other data relevant to future health, and place evidence about the probability of future outcomes, and how to improve those outcomes, at the forefront of clinical thinking and decision-making. Such a model has the potential to reduce or avoid overdiagnosis [8,9] and to promote incorporation of quantitative estimates of future outcomes into shared decision-making with patients in clinical practice [6]. We also discuss potential downsides and limitations of a shift to prognosis-based clinical practice.

## Discussion

### A useful diagnosis is defined by patient prognosis

Diagnosis classifies sick people into groups defined by disease and pathology [10]. The frameworks to explain illness have expanded from pathoanatomical to physiological-biochemical-psychological and, more recently, genetic-molecular models, but the basic concept of diagnosis has not changed. Diagnosis provides clinicians with the means to organise and interpret a range of information provided by patient symptoms, signs, tests, and investigations as the basis for decision-making.

The importance of diagnosis seems most obvious when there is an available treatment which works by directly targeting a specific disease.

Example: *The primary care physician faced with the common clinical problem of a child with fever is concerned not to miss rare but serious diagnoses needing specific treatments. A correct diagnosis of meningococcal meningitis will dictate life-saving, targeted antibiotic treatment. Also, correctly classifying the majority of children with fever who have self-limiting infections will underpin appropriate reassurance, and avoid antimicrobial therapy and potentially harmful specialist investigations.*

Diagnosis and disease mechanisms inform the decisions in this example, yet patient prognosis is the underlying concern. The concern is firstly to correctly identify the few children who, unless they receive urgent treatment targeted at a specific disease, have a high likelihood of poor outcome (disease diagnosis being a highly effective way to improve prognosis in these individuals) and, secondly, to allow the many children with fever who have a high likelihood of a good outcome to recover without disease-targeted interventions.

The science of diagnosis recognises the uncertainty clinicians face as they attempt to classify people with and without disease. In primary care, this drives the search for optimal combinations of symptoms and signs to identify and select patients with high probability of the target condition to undergo further tests. The usefulness of this strategy is judged by prognosis – are outcomes improved in those selected for testing, and is it safe to avoid tests in persons with low disease probability because their prognosis would not be altered by the test?

Example: *Urinary tract infections in children present in ways which overlap with many other acute childhood illnesses. The diagnostic challenge is to identify children whose urine contains bacteria so that antibiotic therapy can be rationalised and kept to a safe minimum. Carrying out high quality microbiology on urine samples from all acutely unwell children is difficult and inefficient. Research seeks clinical prediction rules that select children with an increased probability of bacteriologically-positive infection for urine testing* [11]*, using information including sociodemographics and urinary and non-urinary symptoms. The question is whether application of the prediction rule, plus targeted testing and treatment of identified bacteriologically-positive children, improves outcomes for those who are tested and does no harm to those who are not.*

This example illustrates how a stratified process for diagnosis may support clinical decisions, including avoiding unnecessary investigation and treatment in patients with low disease probability. The usefulness of this process is defined by the prognosis of all children engaged in it.

Diagnostic research emphasises the need for evidence that new tests improve outcomes before adoption in practice [12]. This can be done by linking advances in disease detection with existing evidence for treatment efficacy, or by demonstrating that the new test lowers costs or improves safety. However, it may be necessary to evaluate whether the new diagnostic process changes decision making, improves outcomes in persons classified with the disease, and avoids unnecessary treatment in persons without the disease [13]. The justification for novelty in diagnostic practice is whether it improves patient prognosis. Evidence for this may be lacking.

Example: *A new source of information about suspected coronary heart disease is cardiovascular magnetic resonance imaging. Medical imaging is the fastest growing physician-ordered service in the US Medicare system* [14]*. The most important indicator for cardiovascular imaging in Europe is suspected coronary artery disease and myocardial ischaemia* [15]*. The safety of cardiovascular imaging and its potential to change the diagnosis are established, but whether its use improves patient outcomes has not been addressed, although trials are underway* [16]*. Evidence is needed since* “…as an imaging community we have failed to demonstrate the added value of cardiac imaging in terms of improved quality of care or improved outcomes” [17]*.*

This demand for prognostic evidence of improved outcomes when evaluating new diagnostic information poses substantial challenges of feasibility for the necessary research, especially for studies of long-term impact and cost; this is a limitation on the prognostic model. Information science, and its expanding reservoir of data linked to patient outcomes, will need to drive novel methods to address these questions such as modelling of long-term outcomes by combining data from cross-sectional diagnostic and short-term effectiveness studies.

### Prognosis identifies overdiagnosis

In all three examples above, the usefulness of diagnosis is defined by evidence about patient prognosis, but the clinical process remains focused on disease. The assumption is that identifying individuals with disease optimises their outcome. This assumption may not always be justified.

In the past, disease diagnosis often occurred without effective treatments or any evidence that diagnosis changed outcomes. Even now the culture of ‘diagnosis as an end in itself’, without evidence of its prognostic or practical value for patients, may at best be unnecessary and at worst do harm. There is increasing concern about such ‘overdiagnosis’, in which a pathological lesion or state is identified, and the patient is defined as having a disease, in the absence of any evidence that this state either leads to a poor outcome or defines a pathway of investigation or treatment that clearly advantages the patient. Evidence is accruing that overdiagnosis is not only inefficient in its creation of unnecessary health care, but harmful in the effects which the investigations and treatments generated can have on patients [8].

Example: *A patient presenting with mild urinary symptoms has prostate cancer diagnosed by a test and histopathology. The grading of his cancer places him at one end of the spectrum of risk of future poor outcome, with a low probability that he will die prematurely and evidence that surgical treatment would not alter this probability* [18]*. Furthermore, if surgically treated, there is a risk of undesirable outcomes such as reduced genito-urinary function. The evidence base to inform clinical, personal, and policy decisions needs to show how use of diagnostic tests to identify and classify prostate cancer links to outcomes with and without treatment* [8,9]*, i.e., decisions should be informed by evidence about patient prognosis. We cannot assume the pursuit of diagnosis and disease is beneficial in the absence of evidence about future outcomes.*

Overdiagnosis flourishes in the vacuum created by a culture of ‘underprognosis’, i.e., lack of critical enquiry, information, or evidence about the likely future benefits or harms of identifying a condition as an abnormal disease state. A prognostic framework for clinical practice would help to resist evidence-free diagnostic novelty. Prognostic evidence highlights when overenthusiastic search for pathology leads to irrelevant treatments and needless anxiety, such as disc anomalies on MRI of the spine [19], but can reassure people who need neither active intervention nor a diagnosis, and identify those in whom diagnosis does guide decisions that improve outcomes.

Concerns about overdiagnosis are often generated by screening programmes which diagnose early or latent disease in healthy people. There is debate, for example, about how much breast cancer screening programmes reduce premature mortality, and to what extent the nature and rate of adverse consequences are acceptable, i.e., how population prognosis changes as a result of a screening programme [20]. Overdiagnosis of lesions that do not confer poor prognosis or affect future outcomes is an important adverse consequence of breast cancer screening because of the implications of unnecessary anxiety, investigation, and treatment in patients with such lesions.

### Patient prognosis is determined by more than disease diagnosis

The traditional model of clinical practice assumes that prognosis is inferred only after the diagnosis has been made – presence or absence of disease determines prognosis. However, in the absence of effective treatment, clinicians have always understood that prognosis can be highly variable in persons with a particular diagnosis.

Example: *A physician, working during a typhoid outbreak in the UK in the 1930’s, provided care for the many who recovered and the few who did not* [21]*. He wrote* “A patient with typhoid fever usually inclines to recovery: it is a natural proclivity in one with the disease”*. Diagnosis characterised the sick group but was less important to the people involved than knowledge that most were likely to get better.*

The science of prognosis is concerned with improving the precision, accuracy, and usefulness of measures of likely future outcomes. Modelling an individual’s prognosis can draw on the full range of relevant and available information, both clinical and non-clinical. In the diagnostic model, this may appear as an interaction of disease with non-disease factors in determining outcome such as the influence of psychological health on surgical outcomes. Prognosis offers an alternative starting point with wider incorporation of factors relevant to patient outcomes than diagnosis alone.

Example: *The potential benefit of early diagnosis of type 2 diabetes was investigated in persons identified from primary care records as being at risk of the condition* [22]*. Outcomes were compared between groups invited and not invited for diabetes screening. Management of persons diagnosed with diabetes in the screened group focused on improving prognosis by attempting to reduce their risk of future cardiovascular disease, targeting blood pressure, and cholesterol as well as blood glucose. Ten-year mortality was similar in the screened and unscreened groups. One explanation for this finding was that systematic cardiovascular risk factor management in the screened population was only offered to persons diagnosed with diabetes rather than to everyone invited for screening.*

It is difficult to avoid the conclusion in this example that the focus on diagnosis has obstructed a coherent approach to improving outcomes for people at elevated risk of future cardiovascular events. A focus on improving prognosis regardless of ‘diagnosis’, with blood glucose as one contributor to the probability of poor outcomes, integrated with other risk measures to derive estimates of individual prognosis, would provide a less selective, more productive approach to improving health outcomes. Such an approach may be particularly relevant for patients with multiple health problems.

Example: *Although a person with multiple diseases will benefit from optimal care for each separate condition by disease-based specialists, the multimorbidity state itself contributes to poor prognosis, for example a higher probability of unplanned hospitalisation, and outcomes are improved if there is additional integrated care from generalists* [23]*. However, much multimorbidity concerns risk-based measures (blood sugar, kidney function, blood pressure), which are more usefully considered as continuous variables rather than disease states* [6]*. In constructing prognostic models to support decision-making for people with multimorbidity, such biological measures can be integrated with subjective measures, such as mood state, pain severity, and mobility limitation, to create quantitative estimates of prognosis to inform care for people with multiple long-term conditions.*

Traditional disease-based classification systems are being challenged by the quest for new ways to classify persons with multimorbidity and to incorporate new information about health, such as genomics, into such systems [24]. This new information is undermining the idea that medicine only starts when there is a diagnostic label. Biomedical diagnoses have also traditionally encouraged isolated disease-based measures of outcome, such as normal blood glucose, to assess the success of health care. The acceptance that patient-focused measures, such as improved or maintained social participation, are realistic and desirable outcomes of health care for patients with long-term conditions [25] is subverting the idea that good prognosis is only judged by disease cure.

### Not “have you got it or not?” but “how much have you got?”

Clinical decisions are often dichotomous (does this person have something serious or not? should this patient be allowed to drive or not?). Diagnosis as “either you have it or you don’t” (is it a heart attack or not?) aligns with such yes/no decisions, but the diagnostic process itself is often more probabilistic and uncertain – a series of decisions, guided at each stage by the changed probability of a diagnosis being present or not and designed to gradually reduce uncertainty [26]. However, this process still assumes there is an underlying dichotomous disease state (yes or no); this assumption may be flawed.

The underlying ‘disease’ is often a continuous distribution of probability for future health states. Diagnosis is then not “have you got it?” but “how much of it have you got?” Gale [27], for example, has argued that there is no single pathology underlying diabetes, and that its identification and diagnosis subsumes much heterogeneity, given blood glucose is a continuously distributed variable. Vickers et al. [6] highlight that many such risk variables are artificially dichotomised and treated as disease states rather than as sources of information about probability of future events, which provide a quantitative estimate of individual risk for particular outcomes.

It can be argued that diagnosis does incorporate variability under the concept of disease severity. The continuous distribution of blood pressure, for example, aligns with severity of the ‘disease’ (or unhealthy state). The diagnostic paradigm, however, focuses on classification and misclassification of ‘hypertension’. The prognostic approach more naturally accommodates the idea of severity by relating increasing levels of blood pressure to contrasting outcomes.

A potential downside of the prognostic approach is whether clinical models in the real world can incorporate the continuous nature and variability of risk of future avoidable outcomes into decision making. Calculation of prognostic likelihoods and treatment responsiveness can combine all relevant information, but validation and translation of prognostic models into practice often relies still on categorisation (high-medium-low risk of a particular outcome) to drive stratified care [28,29]. Evidence about the clinical usefulness of such new categorisations is essential – prognostic classification for its own sake should not replace diagnosis for its own sake [30]. Nor should individual patient prognosis be a static classification in time – it needs to be updated with recent predictor values, or a profile of values over time, to reflect how clinical practice works in the real world by using new information and repeat consultations to modify treatment.

There are practical challenges to introducing an information-rich prognosis framework for a real-world clinical practice already struggling with volumes of guidelines, decision-aids, and protocols of care. Technological and statistical advances to make calculation and presentation of prognostic information more accessible and research into ways in which health care professionals and patients can better assimilate, use, and share prognostic information could help to meet these challenges.

### The pros and cons of labelling people

Diagnosis and disease do not represent unchanging truth. Historians and sociologists have described how apparently robust diagnostic entities, such as coronary heart disease, conceal a history of shifting boundaries and definitions [10,31,32]. These shifts reflect not only scientific advance and expanding knowledge (e.g., the identification of *Helicobacter* as a cause of peptic ulcer), but also the recurring need to organise medical knowledge in practical and pragmatic ways to aid clinical and public health decision-making. However, it is equally clear that such shifts are taken up, shaped, and driven by professional ambition, pharmaceutical industry interests, and the commercial drive for profit, and that they influence and are influenced by changing social context and cultural norms, for example, changes in mental illness classification. “*Even US law now recognizes that disease is no longer a unique collection of symptoms equalling a given condition, but rather a constellation of current symptoms, previous exposures, and future potential manifestations, all of which make the art of diagnosis even more precarious*” [33].

Particular challenges occur when no pathological explanation for the patient’s symptoms is currently available.

Example: *Chronic fatigue syndrome is a ‘contested diagnosis’* [31]*. The drive for pathological explanations led to its initial characterisation in the UK as myalgic encephalomyelitis, with little evidence that such a pathological inflammatory process was responsible for the symptoms. The failure of clinical science to identify a mechanism to support the diagnosis means that patients with chronic fatigue often perceive their symptoms are not believed. The outcome of patients with symptoms of persistent fatigue, however, is of high relevance, regardless of the biomedical status of the syndrome.*

Whilst biomedical science often ignores such problems, patients believe biomedicine could and should come up with the answer [32]. Prognostic classification provides a practical way forward. Modifiable patient characteristics that contribute to poor prognosis in chronic fatigue (e.g., low activity level, depression, insomnia) provide targets for intervention in the absence of a definitive biomedical explanation. Targeted exercise programmes, for example, improve prognosis in persons at risk of persisting problems [34].

Diagnosis, however, has functions other than revealing pathological truth. A diagnostic label provides the patient with meaning and value for symptoms regardless of whether these have a biomedical explanation [31]. Diagnosis legitimates the sickness state, and gives access to support and benefits [32]. Prognostic statements may not provide the same immediate value as a diagnostic label, and some patients may be more interested in ‘have I got high cholesterol?’ than in the language of risk. A culture is needed in which clinicians and patients can work together on improving outcomes in the absence of the apparent certainty provided by a diagnostic label.

A hazard of applying a prognostic model in clinical practice relates to the very thing it is designed to reduce, namely over-medicalization of daily life. We have discussed how excessive diagnostic zeal in the absence of improved outcomes for the patient, and the wish to find a pathology to explain every symptom, and the application of diagnostic labels to asymptomatic risk factors, may all lead to ineffective and inefficient care, and harmful side-effects for the patient. The potential for a prognostic model of care to solve these problems has to be weighed against the possibility that such a model may create its own version of over-medicalization. Aronowitz points to healthy individuals locked rather fearfully into long-term surveillance of risk markers, believing this is the way to ensure continuing good prognosis, even if their risk of death and other adverse outcomes is low [35]. The anxieties, and unnecessary and inefficient health care, and commercial and professional interests involved look remarkably similar to those associated with unevidenced diagnostic excess. The resolution lies in demanding high quality evidence of what is and is not useful for improving outcomes, i.e., pursuit of the best prognostic evidence as the scientific basis for resisting excessive medicalization.

### Prognosis provides a natural framework for modern clinical practice

Disease diagnosis is a crucial component of modern medicine but fails to provide a sufficient framework for a modern clinical practice which must incorporate variability in individual patient risk of different outcomes, influences on patient outcome which extend beyond disease, and avoidance of harm; prognosis provides such a framework. Clinicians often think in terms of prognosis, especially the primary care physician who may start by judging if the patient is going to get better or not [36]. Decisions about individual patients in primary care are informed by available evidence about likely future outcomes, and a clinician’s own judgement on likely outcome has prognostic value and helps to guide decision-making [37]. Shared exploration and understanding between clinician and patient of which outcomes are wanted or needed, achieved through patients being able to voice their own priorities and goals for care and treatment in the consultation, supports a prognostic framework for the clinical encounter, particularly for the patient with long-term conditions and multimorbidity [38].

Example: *Evidence that clinicians and patients can integrate disease-based explanation within a broader framework of prognosis is provided by back pain. Primary care practitioners undertake initial triage in a diagnostic framework to identify rare underlying conditions which have a poor immediate prognosis unless treated (e.g., cord compression from a tumour). Once these are excluded, the task diverts from diagnosis and considers the clinical problem as the risk of poor long-term outcomes (work loss, persistent pain). Activity limitation, psychological distress, and capacity to cope are used to classify people into prognostic categories that drive treatment decisions* [39]*. The many at low risk of a poor outcome are managed without referral or investigation, whereas more intense care is targeted at those with poorer prognosis. This exemplifies the principle of ‘stratified care’. Use of this prognostic approach to select back pain patients for different treatment programmes was effective and cost effective in a randomised controlled trial* [40]*.*

Such personalised medicine is likely to herald preferential expansion of prognostic modelling of individual risk for future health outcomes over new diagnostic tests of current disease status. However, research to inform and justify a prognostic model of clinical practice is crucial, including the important uncertainties about the application of this model represented by clinical, patient, and public understanding of risk and probability.

## Summary

We propose that prognosis can now provide the framework in which clinicians and researchers organise evidence and information to support decisions about management. This extends calls for a risk-centred approach to many syndromes and chronic conditions [6] and parallels proposals that public health should be organised around achievable outcomes rather than disease categories [41]. Such a framework shifts the focus of clinical practice to improving outcomes for patients in their total biological, psychological, and social environment and away from an exclusive and narrow focus on underlying disease as the determinant of outcome.

Although biomedical diagnosis is often a crucial driver of treatment selection, especially in acute life-threatening illness such as infection and trauma, diagnostic labelling and subgrouping for many ill people is about providing prognostic information to support decisions about targeted individual long-term care. A broader prognostic framework could integrate such information with evidence about other pathophysiological, psychological, behavioural, and environmental factors that affect patient outcomes, to avoid overdiagnosis and support doctors and their patients in probability-based decision-making in clinical practice.

Prognosis is not a panacea for all the problems we have discussed in relation to diagnosis, and there must be continuing debate about the benefits, value, limits, harms, and costs of medicalization and medical care. However, because prognostic classification incorporates a much wider range of information than diagnosis and disease factors alone, and because such information is expanding rapidly in volume and availability [42], prognosis can provide a realistic, practical, and useful framework for clinical and public health practice.
